# *Aedes aegypti* D7 Saliva Protein Inhibits Dengue Virus Infection

**DOI:** 10.1371/journal.pntd.0004941

**Published:** 2016-09-15

**Authors:** Michael J. Conway, Berlin Londono-Renteria, Andrea Troupin, Alan M. Watson, William B. Klimstra, Erol Fikrig, Tonya M. Colpitts

**Affiliations:** 1 Foundational Sciences, Central Michigan University College of Medicine, Mt. Pleasant, Michigan, United States of America; 2 Department of Pathology, Microbiology and Immunology, University of South Carolina School of Medicine, Columbia, South Carolina, United States of America; 3 Center for Vaccine Research and Department of Microbiology and Molecular Genetics, University of Pittsburgh, Pittsburgh, Pennsylvania, United States of America; 4 Section of Infectious Diseases, Department of Internal Medicine, Yale University School of Medicine, New Haven, Connecticut, United States of America; 5 Howard Hughes Medical Institute, Chevy Chase, Maryland, United States of America; University of Florida, UNITED STATES

## Abstract

*Aedes aegypti* is the primary vector of several medically relevant arboviruses including dengue virus (DENV) types 1–4. *Ae*. *aegypti* transmits DENV by inoculating virus-infected saliva into host skin during probing and feeding. *Ae*. *aegypti* saliva contains over one hundred unique proteins and these proteins have diverse functions, including facilitating blood feeding. Previously, we showed that *Ae*. *aegypti* salivary gland extracts (SGEs) enhanced dissemination of DENV to draining lymph nodes. In contrast, HPLC-fractionation revealed that some SGE components inhibited infection. Here, we show that D7 proteins are enriched in HPLC fractions that are inhibitory to DENV infection, and that recombinant D7 protein can inhibit DENV infection *in vitro* and *in vivo*. Further, binding assays indicate that D7 protein can directly interact with DENV virions and recombinant DENV envelope protein. These data reveal a novel role for D7 proteins, which inhibits arbovirus transmission to vertebrates through a direct interaction with virions.

## Author Summary

Dengue virus (DENV) is transmitted to humans by *Aedes aegypti* during the blood feeding process. During blood feeding, DENV and saliva proteins are inoculated into human skin. D7 proteins are prevalent and immunogenic proteins present in *Ae*. *aegypti* saliva, and assist the blood feeding process by scavenging biogenic amines. Previous data suggests that antibodies against D7 protein from *Culex spp*. can increase West Nile virus infection. We hypothesized that D7 proteins may also have antiviral activity. Here, we show that recombinant *Ae*. *aegypti* D7 protein can inhibit DENV infection *in vitro* and *in vivo*, and that D7 can bind to DENV virions.

## Introduction

Dengue virus (DENV) is a mosquito-borne arbovirus that is transmitted primarily by the species *Aedes aegypti*. The global burden of DENV has grown dramatically in the last few decades [1, 2]. We now expect approximately one hundred million clinically recognized cases of disease each year. Targeted therapeutics do not exist. Fortunately, conventional vaccines are in development and regulatory approval has been granted in a few countries. Development of a conventional DENV vaccine has been difficult due to the co-circulation of four serotypes [3, 4]. It is critical that conventional vaccines elicit robust antibody titers to avoid antibody-dependent enhancement, which occurs during a sub-neutralizing response. It is theoretically possible to target mosquito saliva or midgut proteins to block either transmission or acquisition of DENV [3, 5, 6]. This strategy would not be subject to antibody-dependent enhancement or viral genetic drift.

*Ae*. *aegypti* saliva contains over one hundred unique proteins that have been classified as D7 proteins, phosphatidylethanolamine binding proteins, odorant and juvenile hormone binding proteins, serpins and other protease inhibitors, a sialokinin vasodilator, nucleotidases, serine proteases, sugar digestion related proteins and other enzymes, lectins, angiopoietins, anti-microbial proteins and peptides, mucins and peritrophins, antigen 5 proteins, and many more proteins of unknown function [7–11]. Functional data is not available for the majority of these proteins, although it is expected that the saliva of all hematophagous arthropods have anti-coagulant, anti-platelet, and vasodilatory activities. It is also likely that saliva proteins serve to reduce host inflammation and prevent infection.

In addition to the normal physiological roles of hematophagous arthropod saliva, many vector-borne microorganisms have enhanced fitness in the presence of arthropod saliva. Arthropod saliva can enhance infectivity of West Nile virus, DENV, Rift Valley fever virus, and Powassan virus, among others [5, 12–18]. The exact mechanism of saliva-mediated infectivity enhancement is not known, although prior literature suggests that saliva proteins may locally modify the immune system in favor of arbovirus replication and/or stimulate dissemination by enhancing migration of target cells to draining lymph nodes [3]. Interestingly, individual saliva components can have inhibitory activities against arbovirus infection. For instance, the collagen-binding protein aegyptin decreased DENV infection *in vivo* [19]. Additionally, previous literature showed that vaccination of mice with a recombinant D7 protein from *Culex spp*. enhanced mortality in a West Nile virus mouse model, suggesting that D7 protein may be inhibitory *in vivo* [20]. Structural studies suggest that D7 proteins can simultaneously bind biogenic amines and cysteinyl leukotrienes, which is likely involved in preventing the host inflammatory response [21, 22]. Prevention of the host inflammatory response may reduce influx or activation of target cells.

Our previous work relied on high performance liquid chromatography (HPLC) to fractionate *Ae*. *aegypti* salivary gland extracts (SGEs) [5]. HPLC fractions were tested to see if they had virus enhancing or blocking activities *in vitro*. Here, we identified a number of fractions that inhibited DENV cell binding and then pooled these fractions for downstream tandem liquid chromatography tandem mass spectrometry (LC+MS/MS) analysis. D7 proteins were the most abundant proteins in the inhibitory fractions. We synthesized a recombinant D7 protein and found that it inhibited DENV infection *in vitro* and *in vivo*. Additionally, the D7 protein interacted directly with DENV virions and recombinant envelope protein. These data support a model whereby D7 proteins inhibit DENV infection through two independent mechanisms: (i) direct binding and neutralization of DENV virions, and (ii) inhibition of immune cell infiltration or activation, which reduces the number of permissive target cells. Characterization of virus-vector-host interactions at the transmission interface will further development of arthropod-based therapeutics and transmission-blocking vaccines.

## Methods

### Mosquito rearing and saliva material collection

*Aedes aegypti* were provided by staff at the Connecticut Agricultural Experiment Station. Mosquitoes were maintained in a sugar solution at 27°C and 80% humidity according to standard rearing procedures. Salivary glands and saliva were isolated as described previously [5]. Salivary gland extracts were prepared by placing 100 salivary glands in 100 μl sterile phosphate-buffered saline (PBS), freeze-thawing by placing on dry ice three times, and then removing insoluble debris by centrifugation at 5,000 × *g* for 10 min. Saliva was isolated using the immersion oil technique.

### Cell culture and virus stocks

Mouse embryonic fibroblasts (MEFs) and a human monocyte-like (U937) cell line from the American Type Culture Collection were maintained in Dulbecco's modified Eagle medium (DMEM) containing 10% fetal bovine serum and antibiotics at 37°C with 5% CO_2_ (Gibco). C6/36 cells were maintained in DMEM containing 10% fetal bovine serum, tryptose phosphate, and antibiotics at 30°C. DENV2 was passaged in C6/36 cells. DENV2 New Guinea C strain was obtained from the Connecticut Agricultural Experiment Station and C6/36 cells were a kind gift from Erol Fikrig. Approximately 1 × 10^5^ genome equivalents (GE) were used for *in vitro* infections of MEFs and U937 cells.

### HPLC fractionation and LC+MS/MS

One hundred salivary glands were dissected from female *Ae*. *aegypti* and placed in 100 μl PBS. The sample was freeze-thawed three times at −80°C, and insoluble debris was pelleted by centrifugation at 5,000 × *g* for 10 min. The supernatant was reserved. SGE was either processed directly for LC+MS/MS analysis or fractionated by high-performance liquid chromatography (HPLC) on a nonporous reverse-phase column with a TFA buffer system into 80 100-μl fractions. Ten μl of each fraction was diluted into 90 μl PBS and used as SGE treatments for *in vitro* SGE-mediated cell binding assays as stated below. The remaining 90 μl from inhibitory fractions 31–49 were pooled and submitted for liquid chromatography tandem mass spectrometry (LC+MS/MS) analysis.

Proteins were digested with trypsin and analyzed using LC+MS/MS on a Thermo Scientific LTQ-Orbitrap XL mass spectrometer using Waters nanoACQUITY ultra-high-pressure liquid chromatographs (UPLC) for peptide separation. MS/MS spectra were searched in-house using the Mascot algorithm for uninterpreted MS/MS spectra after using the Mascot Distiller program to generate Mascot-compatible files. An *A*. *aegypti* database was used for searching. The Keck Biotechnology Resource at Yale University performed both HPLC and LC+MS/MS.

### *In vitro* cell binding assay

MEFs were seeded at 25,000 cells/well in 48-well plates and grown to approximately 70% confluence overnight. Medium was aspirated, and cells were washed with PBS. Ten μl of HPLC fractions were diluted in a total volume of 100 μl PBS and inoculated into cells at room temperature for 10 min. Saliva material was removed, and then Approximately 1 × 10^5^ GE of DENV2 was inoculated into cells in a total volume of 500 μl for 1 h at 37°C. Unbound virus was then removed, and fresh medium was added. Infections progressed for up to 18 h. Total RNA was extracted using RNeasy kits (Qiagen).

For analysis of relative DENV vRNA, total RNA was harvested using RNeasy kits (Qiagen). Amplification of both the viral target and reference gene target was performed using a duplex format in 0.2-ml, 96-well PCR plates (Bio-Rad) with a total reaction volume of 25 μl. Reverse transcription and quantitative PCR (RT-qPCR) were performed in the same closed tube with 250 ng of total RNA per reaction using the Quantitect RT-PCR kit (Qiagen).

All primers were used at a final concentration of 4 μM and were synthesized by the Keck Facility at Yale University. DENV2 vRNA was amplified using F 5’ CCACTGCCTCTGGAAAACTC 3’ and R 5’ GTACCAGCACCCATCCTCAC 3’ primers. Primers were developed using Gene Link Software (OligoAnalyzer 1.2 and OligoExplorer 1.2). All RT-qPCRs were performed using an iQ5 machine (Bio-Rad). Cycling conditions were 50°C for 30 min (reverse transcription) and 95°C for 15 min, followed by 42 cycles of 94°C for 15 s and 54.5°C for 1 min. Relative quantities of viral target cDNA were determined using REST software.

### Cloning

*Ae*. *aegypti* total cellular RNA was converted to cDNA using random primers and the Superscript III First-Strand Synthesis System (ThermoFisher). Long form D7 protein (AAEL006424) was amplified using F 5’ GGAGGTACCGATGAAGCTGCCTCTATTACTCGCAATAGTTAC 3’ and R 5’ GGAGCGGCCGCAATTGTGGACACTGTTTACCGTCG 3’ primers and cloned into the pMT/BiP/V5-His A plasmid via BamHI and NotI restriction sites. pMT/BiP/D7/V5-His A and pCoHygro plasmids were transfected into S2 cells using the calcium phosphate method according to manufacturers’ instructions (ThermoFischer) and a stable cell line was generated through hygromycin selection. D7 synthesis and secretion was induced by treating cells with copper sulfate according to manufacturer’s instructions (ThermoFisher). D7 expression was confirmed by Coomassie Blue gel staining and Western blot using an anti-6 His antibody. Supernatants were also harvested from uninduced S2 cell supernatants and used as negative controls.

### *In vitro* infectivity assay

D7 protein was purified using HisPur Cobalt Spin Columns (Thermo Scientific/Pierce, MA) according to manufacturer instructions. There were a total of 3 washes and 3 elutions. Samples were stored at -80°C until use. The U937 cell line (ATCC, VA) was used for the *in vitro* infection studies. The cells were grown at 37°C and 5% CO2 in DMEM supplemented with 10% fetal bovine serum (Gemini, CA), and 1% penicillin-streptomycin. D7 was used in 1/10, 1/100, and 1/1000 dilutions in complete media for final concentrations of 8, 0.8, and 0.08 ng/mL, respectively. Dilutions were added for pretreatment of cells in a total volume of 250 μL. Cells were then incubated for 1 hour at 37°C and then DENV was added to cells at a multiplicity of infection (MOI) of 1.0. For simultaneous treatments, ten-fold dilutions of D7 were mixed with DENV2 and these mixtures were pre-incubated for 1 hour at 37°C. D7-DENV2 mixtures were then inoculated onto cells. Unbound virions were removed by washing after 1 hour and then cells were incubated for 24 hours at 37°C. RNA was isolated from cells and qRT-PCR performed as described above. DENV2 vRNA was amplified using Forward: 5’ CAG ATC TCT GAT GAA TAA CCA ACG 3’ and Reverse: 5’ CAT TCC AAG TGA GAA TCT CTT TGT CA 3’ primers. Human B2M RNA was amplified using Forward: 5’ CTC CGT GGC CTT AGC TGT G 3’ and Reverse: 5’ TTT GGA GTA CGC TGG ATA GCC T 3’ primers.

### Ethics statement

Animals were maintained and procedures were performed in accordance with the recommendations in the Guide for the Care and Use of Laboratory Animals of the National Research Council. Protocol 14051879 was approved by the University of Pittsburgh's IACUC committee. Approved euthanasia criteria were based on weight loss and morbidity.

### *In vivo* infectivity assay

Mice deficient in receptors for type I and type II interferons, (IFNAGR^-/-^, AGB6) were bred under specific pathogen-free conditions. Groups of 4-5-week-old, age-matched, mixed-sex AGB6 mice were inoculated subcutaneously into both rear footpads with 20 μl containing either 10^7^ genome equivalents (GE) of either DENV2 alone or DENV2 plus recombinant D7 protein. DENV2 alone samples contained purified S2 cell supernatant without D7 protein as a vehicle control. Forty-eight hours post-infection, mice were euthanized and left and right foot pads and left and right popliteal draining lymph nodes (DLN) were collected independently. Total RNA was extracted using an RNeasy kit (Qiagen). qRT-PCR was performed by adding equal amounts of RNA into each reaction and data were normalized to the actin reference gene. Mouse actin RNA was amplified using Forward: 5’ GGC TGT ATT CCC CTC CAT CG 3’ and Reverse: 5’ CCA GTT GGT AAC AAT GCC ATG T 3’ primers. Amplification of targets were performed using a duplex format in 0.2 ml, 96-well PCR plates (BIO-RAD) with a total reaction volume of 25 μl. Reverse transcription and quantitative PCR were performed in the same closed tube with 100 ng of total RNA per reaction using the Quantitect RT-PCR Kit (Qiagen).

### Salivary gland extract-DENV virion binding assay

Seventy μg of anti-dengue virus type II antibody, clone 3H5-1 (Millipore) was covalently coupled to 25 μL amine-active resin according to the Pierce Co-Immunoprecipitation Kit manual (ThermoFisher Scientific). One hundred μg purified, formaldehyde-inactivated dengue virus type 2 virions (Microbix) was then mixed with 100 *Ae*. *aegypti* salivary gland equivalents and added to the antibody-containing resin with gentle end-over-end mixing for 2 hr at 4°C. Unbound proteins were washed away and bound proteins were eluted in a final volume of 50 μL. The experimental details are described in the Pierce Co-Immunoprecipitation Kit manual. Eluted proteins were identified by LC+MS/MS analysis as stated above.

### Enzyme-linked immunosorbent assay

High binding 96-well plates were coated overnight at 4°C with a 1:1 dilution of D7 or matrix metalloproteinase protein (MMP; AAEL003012) in coating buffer (R&D systems) for a 0.01mg/mL final concentration. The next day, plates were rinsed twice with PBS and blocked with blocking buffer (1% BSA in 1XPBST) for 1 h at 37. Plates were incubated with 80μL of DENV 2 strain 186881 (2x10^5^ p.f.u) or recombinant DENV Envelope protein (1μg/mL) (L2 Diagnostics, CT) overnight at 4°C. Plates were washed three times with buffer (1X PBST+ 0.01% Tween 20) and incubated with a 1:250 dilution of primary antibody for 1h at 37°C: mouse anti-DENV2 antibody (MAB10226, Millipore) to detect virus and mouse anti-E antibody (MA1-71251, Pierce) to detect envelope protein. After washing plates three times, wells were incubated with 100 μL of a 1:1000 dilution of anti-mouse IgG, HRP-linked antibody (Cell Signaling Technologies) and washed after 1h of incubation at 37°C. Reaction was visualized after incubating with 80 μL of TMB. After 3min the reaction was stopped with stop solution (2M Sulfuric acid) and plates were read at 450nm in a Synergy HT plate reader (Biotek Instruments Inc., VT).

## Results

### Identification of D7 proteins in inhibitory salivary gland extract high performance liquid chromatography fractions

**Fig 1:**
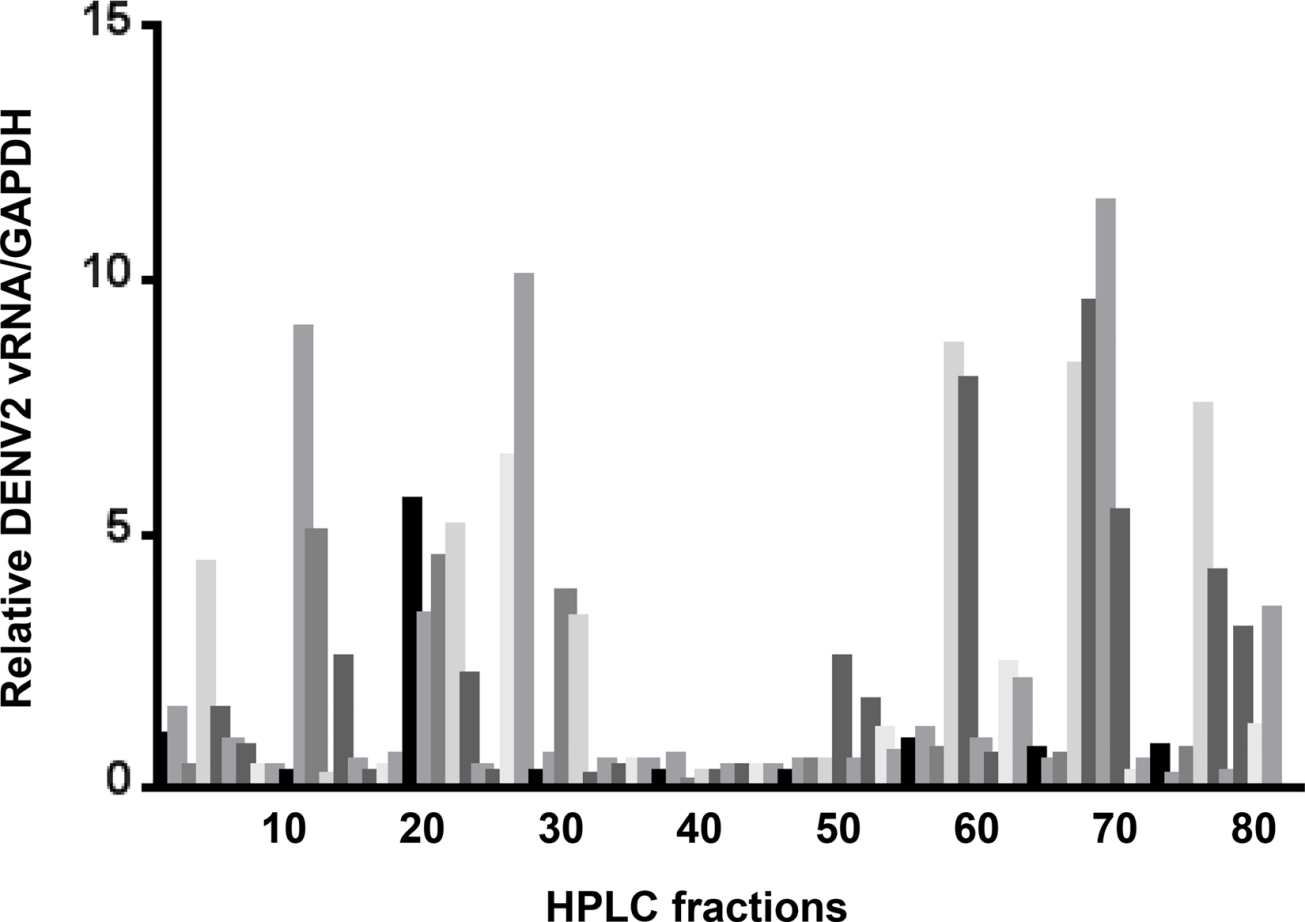
Impact of HPLC-fractionated *Ae*. *aegypti* salivary gland extract on DENV2 cell binding. Individual HPLC fractions were used to pre-treat mouse embryonic fibroblasts prior to inoculation with DENV2. Total RNA was harvested and qRT-PCR was performed 18 hours post-infection. Relative levels of DENV2 were normalized to GAPDH. An untreated control was set to 1.0 and was placed on the far left of the graph (black bar).

We previously showed that pre-treatment of mouse embryonic fibroblasts (MEFs) with salivary gland extract (SGE) increased DENV cell binding [5]. Reverse phase HPLC fractionation and LC+MS/MS analysis was then used to identify fractions of *Ae*. *aegypti* salivary gland extract (SGE) that increased DENV vRNA levels associated with mouse embryonic fibroblasts (MEFs)[5]. During this analysis, we also noted that a cluster of HPLC fractions (i.e., 31–49) inhibited DENV infection (Fig 1). These fractions were pooled and submitted for LC+MS/MS analysis and searched against the NCBI *Ae*. *aegypti* database to identify the most abundant proteins. Multiple proteins were identified with high score values, although long forms of the D7 protein family were the most prevalent (Table 1). A number of these proteins were not predicted as secreted proteins that would be present in saliva. To validate the proteins that are present in saliva and may play a role at the virus-vector-host interface, saliva was harvested from one hundred *Ae*. *aegypti* using the immersion oil technique [23]. Soluble proteins were extracted from immersion oil using phosphate buffered saline and the sample was submitted for LC+MS/MS analysis. We confirmed that 7 proteins in the inhibitory fraction are also present in saliva (Tables 1 and 2). Three of these proteins were either long or short form D7 proteins.

**Table 1:** LC+MS/MS hits from pooled inhibitory HPLC SGE fractions.

Identifier	Comments	Score	Present in saliva
AAEL006424	D7 protein long	2221	Yes
AAEL006417	D7 protein long	2100	Yes
AAEL010235	30 kDa salivary protein	1852	Yes
AAEL002761	Tropomyosin	1023	No
AAEL011949	Transferrin	984	No
AAEL006333	Apyrase	933	Yes
AAEL006096	Gelsolin	898	No
AAEL001005	Calreticulin	725	No
AAEL010228	30 kDa allergen-like protein	710	Yes
AAEL006423	D7 protein short	707	Yes
AAEL003566	Galactose-specific C-type lectin	688	No
AAEL000533	Antifreeze protein	650	No
AAEL012897	Mitochondrial aconitase	621	No
AAEL006406	Hypothetical protein	603	No
AAEL009852	14.5 kDa salivary protein	586	No
AAEL002539	Calponin/transgelin	579	No
AAEL002417	Troponin T isoform 1	564	No
AAEL013528	Peroxiredoxin	467	No
AAEL007394	Hypothetical protein	432	No
AAEL010242	8.7 kDa salivary protein	403	No
AAEL003957	Hypothetical protein	387	No
AAEL003600	34 kDa salivary protein	382	Yes
AAEL013279	Cyclophilin	344	No
AAEL017096	Translation elongation factor EF-1 alpha/Tu	299	No
AAEL013407	Hypothetical protein	293	No
AAEL017096	Lysozyme	280	No

**Table 2:** LC+MS/MS hits from *Aedes aegypti* saliva.

Identifier	Comments	Score
AAEL000732	62 kDa secreted protein	1227
AAEL003182	Serine protease inhibitor (serpin)	738
AAEL005672	Adenosine deaminase	721
AAEL006417	D7 protein long	652
AAEL006347	Apyrase precursor	648
AAEL003600	34 kDa salivary protein	535
AAEL003601	34 kDa salivary protein	430
AAEL002704	Serine protease inhibitor (serpin)	361
AAEL006424	D7 protein long	344
AAEL010235	30 kDa salivary gland protein	336
AAEL009081	56.5 kDa salivary protein	329
AAEL003053	Allergen	302
AAEL006333	Salivary apyrase	286
AAEL000748	62 kDa secreted protein	270
AAEL006485	Inosine-uridine preferring nucleoside hydrolase	210
AAEL010228	30 kDa allergen-like protein	199
AAEL003100	Salivary mucin	181
AAEL000793	Venom allergen	181
AAEL005766	Fructose-bisphosphate aldolase	148
AAEL011197	Actin	132
AAEL003107	Hypothetical	123
AAEL009992	Hypothetical	120
AAEL006423	D7 protein short	107
AAEL000490	Histone H4	82
AAEL009955	Vitellogenin-like protein	73
AAEL006511	Ubiquitin	71
AAEL009993	SGS1	68

**Fig 2:**
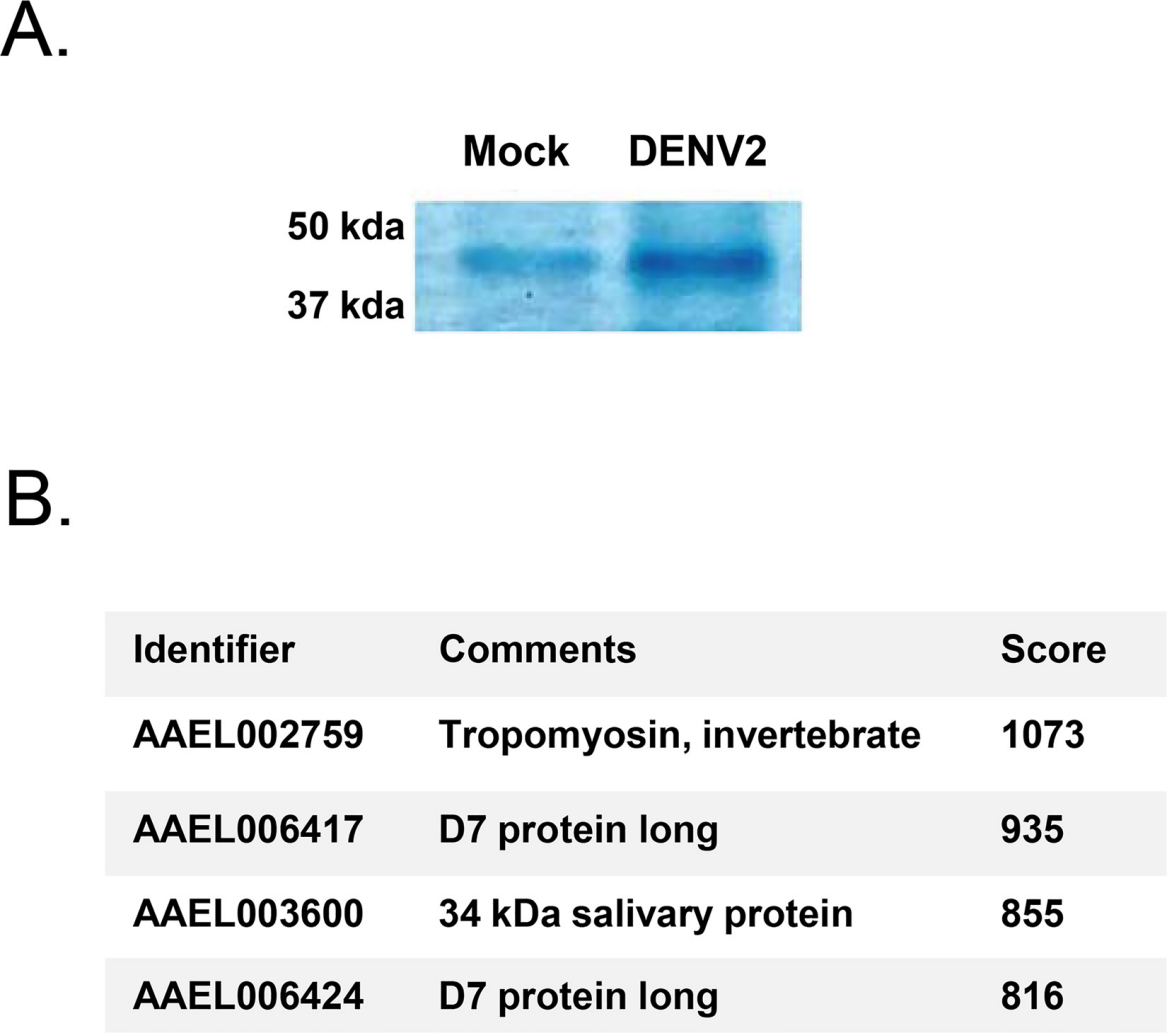
Identification of upregulated *Ae*. *aegypti* salivary gland proteins during DENV2 infection. (A) Section of a Coomassie Blue stained gel containing approximately 1 μg salivary gland extracts from mock-infected and DENV2-infected *Ae*. *aegypti*. A single band was more intense during DENV2 infection. (B) Proteins identified within the DENV2-specific band as shown above by mass spectrometry.

In a separate experiment, we sought to elucidate proteins that are upregulated in salivary glands during DENV2 infection. *Ae*. *aegypti* were either mock or infected with DENV2 via blood feeding using an artificial membrane. Twenty salivary glands were isolated from each group of mosquitoes 14 dpi and equal amounts of protein were loaded on to an SDS-PAGE gel for one-dimensional gel electrophoresis. Gels were stained with Coomassie Blue. Multiple bands were different between mock and DENV2 lanes, but one band appeared significantly darker in the DENV2-infected lane (Fig 2A). The band from the DENV2-infected lane was excised and submitted for mass spectrometry. Only four unique *Ae*. *aegypti* proteins were detected, which included tropomyosin, two long form D7 proteins, and a 34 kDa salivary protein (Fig 2B). We confirmed that both long and short form D7 proteins and the 34 kDa salivary protein are upregulated in *Ae*. *aegypti* salivary glands at the transcriptional level during DENV2 infection by assessing previously published RNA Seq data (Table 3) [11].

**Table 3:** Gene accumulation levels in FPKM in mock and DENV2-infected *Ae*. *aegypti* salivary glands 14 days post-infection.

Identifier	Comments	Mock	DENV2
AAEL006417	D7 protein long	5,869	11,411
AAEL006424	D7 protein long	13,440	28,913
AAEL006423	D7 protein short	2,828	7,982
AAEL006408	D7 protein short	1,960	2,336
AAEL006406	D7 protein short	4,785	7,916
AAEL007394	D7 protein short	1,529	6,718
AAEL003600	34 kDa salivary protein	6,287	14,361
AAEL002759	Tropomyosin, invertebrate	36	11

### Recombinant D7 protein inhibits dengue virus *in vitro* and *in vivo*

**Fig 3:**
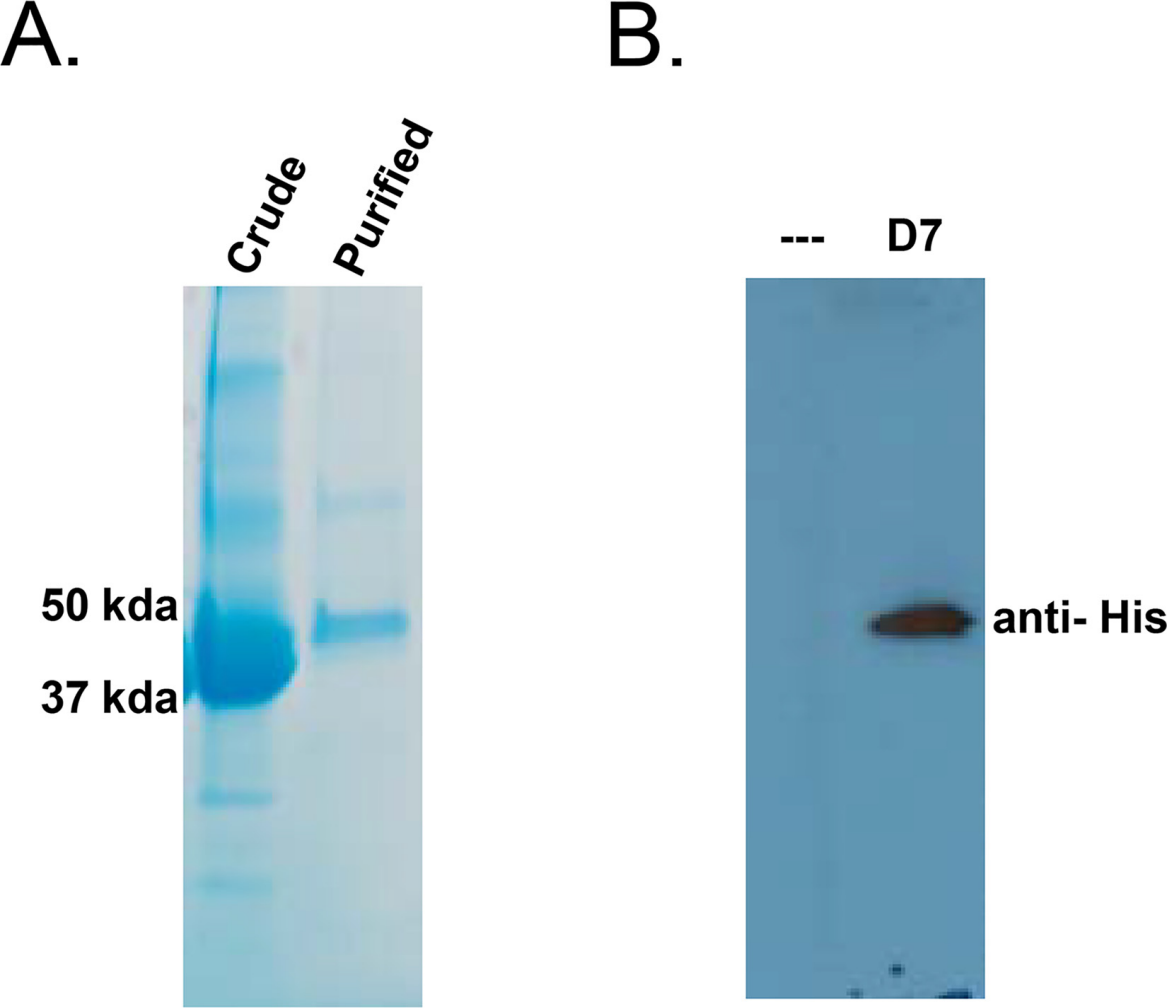
Characterization of recombinant D7 protein. (A) Coomassie Blue gel stain of crude S2 cell lysate containing recombinant D7 protein and D7 protein after purification using a HisPur Cobalt Spin Column. (B) Western blot of uninduced (—) and induced (D7) S2 cell supernatants using an anti-6 His antibody.

**Fig 4:**
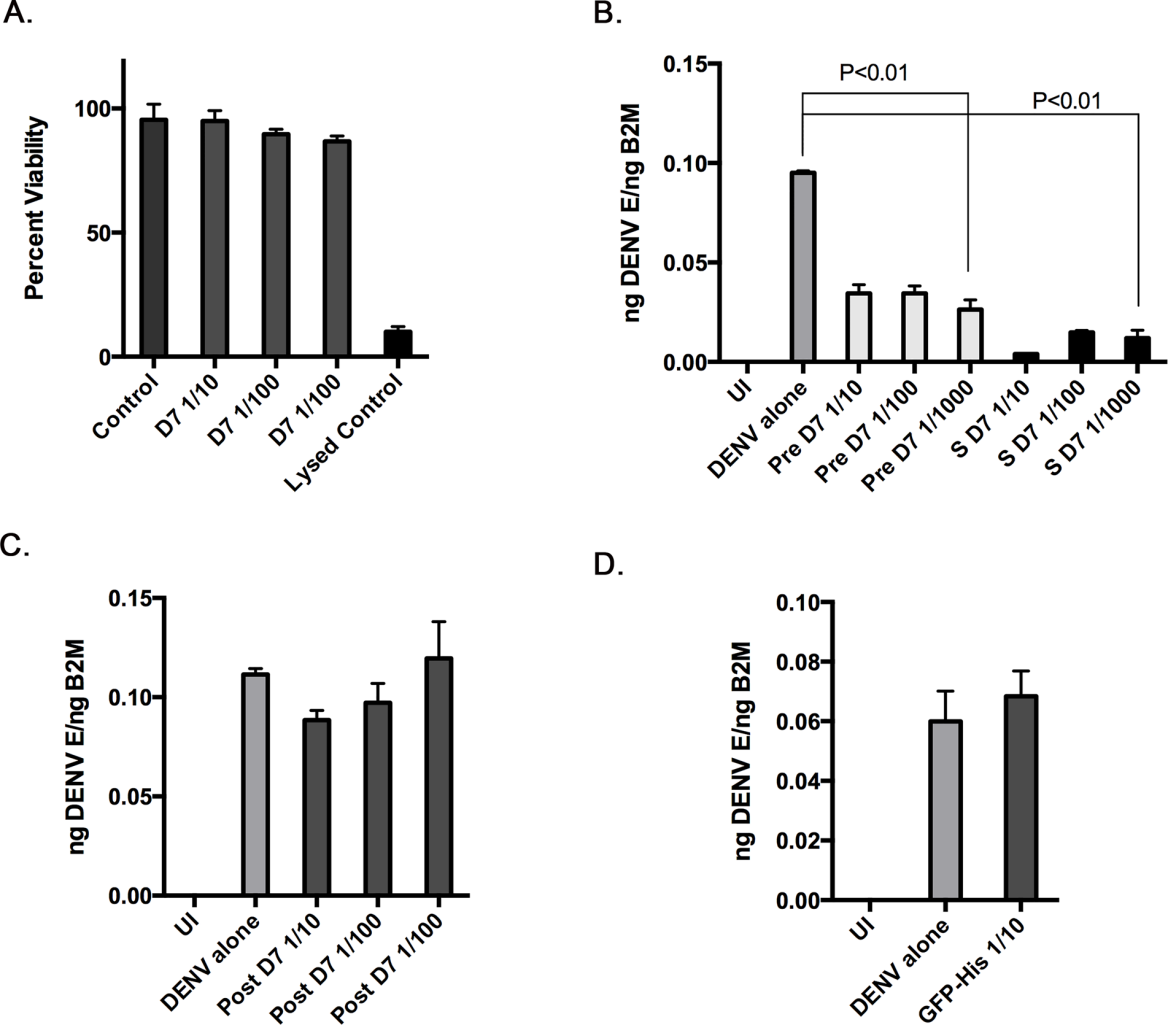
Recombinant D7 protein inhibits DENV2 infection in U937 cells. (A) The Promega CellTox^TM^ Green Cytotoxicity Assay was used to determine the toxicity of ten-fold dilutions of recombinant D7 protein on U937 cells 24 hours post-treatment. (B) U937 cells were either uninfected (UI), infected with DENV2 alone, pre-treated with 10-fold dilutions of recombinant D7 protein followed by DENV2 infection, or co-treated with 10-fold dilutions of recombinant D7 protein with concomitant DENV2 infection. Total RNA was harvested and qRT-PCR was performed 24 hours post-infection. Relative levels of DENV2 were normalized to B2M. (C) U937 cells were either uninfected (UI), infected with DENV2 alone, or infected followed by post-treatment with 10-fold dilutions of recombinant D7 protein. Post-treatment began 24 hpi and lasted 8 hours. Total RNA was harvested and qRT-PCR was performed 48 hours post-infection. Relative levels of DENV2 were normalized to B2M. (D) U937 cells were either uninfected (UI), infected with DENV2 alone, or pre-treated with recombinant Hig-tagged GFP, representing the highest concentration used for D7 protein followed by DENV2 infection. Total RNA was harvested and qRT-PCR was performed 24 hours post-infection. Relative levels of DENV2 were normalized to B2M. Data represent averages of at least 3 independent experiments. Student’s t tests were performed to assess statistical significance between groups.

*Ae*. *aegypti* D7 proteins are multifunctional and have been shown to bind to cysteinyl leukotrienes (cysLT) and biogenic amines with high affinity. Based on the discovery of D7 proteins in inhibitory HPLC fractions, and of increased D7 protein expression in DENV2-infected salivary glands, we hypothesized that D7 proteins may bind to additional substrates with varying affinities, and that these interactions inhibit DENV cell binding and infectivity. To test this hypothesis, a recombinant D7 long form protein (AAEL006424) was produced in S2 cells and harvested from the supernatant at a concentration of 80 ng/mL (Fig 3A and 3B). We determined if D7 protein was toxic to cells by incubating ten-fold dilutions of D7 protein with monocyte-like U937 cells for 24 hours followed by analysis using Promega’s CellTox Green Cytotoxicity Assay. This assay measures membrane integrity that occurs as a result of cell death. Both positive and negative controls were included. None of the dilutions tested were toxic to U937 cells at the time point tested (Fig 4A).

We then tested if D7 protein had antiviral activity *in vitro* by either pre-incubating U937 cells with ten-fold dilutions of recombinant D7 protein for 1 hour at 37°C prior to inoculation with DENV2, or incubating ten-fold dilutions of D7 protein with DENV2 for 1 hour at 37°C prior to co-treatment with D7-DENV2 mixtures. The different treatment strategies were employed to determine if D7 needed to directly interact with virions for activity. Total RNA was extracted 24 hpi and the relative amount of DENV2 vRNA was normalized to a housekeeping gene. Both pre-treatment and co-treatment of recombinant D7 protein and DENV2 significantly reduced DENV2 vRNA levels in U937 cells and there wasn’t a statistical difference between these two treatment groups (Fig 4B). A post-treatment experiment was also performed to determine if D7 inhibited DENV at an early time point during infection. In this experiment, U937 cells were infected with DENV for 24 hours, followed by an 8 hour treatment with ten-fold dilutions of D7 protein. Total RNA was extracted 48 hpi and the relative amount of DENV2 vRNA was normalized to a housekeeping gene. This treatment strategy did not significantly inhibit DENV infection, suggesting that D7 inhibits DENV at an early time point (Fig 4C). We also tested if an irrelevant His-tagged protein could inhibit DENV infection. Recombinant His-tagged GFP was unable to inhibit DENV infection at the highest concentration tested for D7 (Fig 4D).

**Fig 5:**
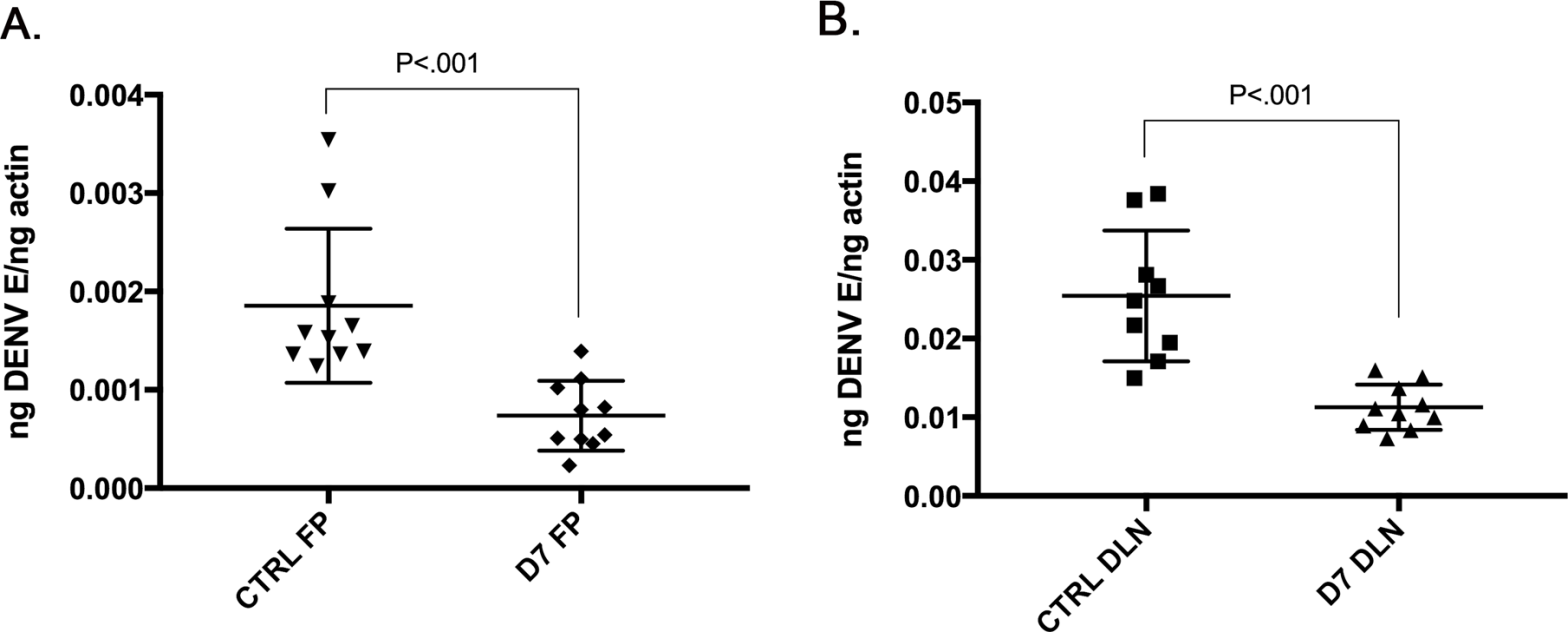
Recombinant D7 protein inhibits DENV2 infection in AGB6 mice. Mice deficient in receptors for type I and type II interferons, (IFNAGR-/-, AGB6) were inoculated subcutaneously into both rear footpads with 20 μl containing either 10^7^ genome equivalents of either DENV2 alone or DENV2 plus recombinant D7 protein. Forty-eight hours post-infection, mice were euthanized and left and right foot pads and left and right popliteal draining lymph nodes (DLN) were collected independently. (A) Total RNA was harvested from footpad tissue and qRT-PCR was performed to assess relative levels of DENV2 vRNA. Relative DENV2 vRNA was normalized to actin gene expression. (B) Total RNA was harvested from DLNs and qRT-PCR was performed to assess relative levels of DENV2 vRNA. Relative DENV2 vRNA was normalized to actin gene expression. 5 mice were present in each group and the data represents a single experiment. Unpaired t tests were performed to assess statistical significance.

To determine if recombinant D7 can inhibit DENV2 infection *in vivo*, we challenged AGB6 mice with DENV2 with and without D7 protein by subcutaneous footpad inoculation. AGB6 mice were chosen because they are highly permissive to DENV2 infection and were a suitable model for early DENV2 replication and dissemination to draining lymph nodes. Briefly, 2 groups of 4-5-week-old, age-matched, sex-matched mice were inoculated into a single rear footpad with 20 μl containing 1 x 10^7^ genome equivalents, DENV2 alone or in combination with approximately 1 ng recombinant D7 protein. In order to test the impact of D7 treatment on early replication and dissemination, RNA was harvested from footpads and draining lymph nodes 48 hpi and qRT-PCR was used to measure the relative levels of DENV2 normalized to an actin reference gene [5]. D7 protein significantly reduced DENV2 vRNA levels in both the footpads and draining lymph nodes 24 hpi (Fig 5A and 5B). DENV2 vRNA amplified at Ct values of 21–24 in footpad samples and 17–19 in draining lymph node samples.

### Recombinant D7 protein directly interacts with dengue virions and envelope protein

Our results suggest that D7 protein can inhibit DENV2 infection *in vitro* and *in vivo*, although it is unclear how D7 protein mediates its antiviral effect. Pre-treatment of U937 cells with recombinant D7 protein led to a significant decrease in DENV2 infection, suggesting that D7 protein may modulate the host cell. Although, co-treatment of D7 protein with DENV2 appeared to be more effective at preventing cell binding and/or infection of U937 cells. We hypothesized that D7 protein can interact with multiple substrates including proteins at the cell surface and viral proteins. To test this hypothesis, we performed binding assays to determine if D7 protein can bind to DENV virions and envelope protein.

First, anti-DENV2 antibody, clone 3H5-1 was covalently coupled to 25 μL amine-active resin in two separate columns. One hundred μg of purified, formaldehyde-inactivated dengue virus type 2 virions was then mixed with 100 *Ae*. *aegypti* salivary gland equivalents and added to the antibody-containing resin and incubated for 2 hr at 4°C. A negative control column containing anti-DENV2 antibody was also prepared with only 100 salivary gland equivalents. Unbound proteins were washed away and bound proteins were eluted. Eluted proteins were identified by LC+MS/MS analysis. Although Mascot scores were low, 8 proteins were identified that were unique and not in the negative control and three of these were present in saliva (Tables 2 and 4). This included the long form D7 protein (AAEL006424). To determine if the proteins we detected were simply correlated with their abundance, we performed LC+MS/MS analysis on *Ae*. *aegypti* salivary gland extracts (Table 5). Although actin and serine protease inhibitor (serpin) had Mascot scores in the top 3%, Mascot data suggested that the remaining proteins were less abundant, and that our co-immunoprecipitation data did not simply represent the most abundant proteins in salivary gland extracts.

**Table 4:** LC+MS/MS hits from SGE/DENV2 binding assay.

Identifier^a^	Comments	Score	Present in saliva
AAEL001951	Actin	137	No
AAEL003601	34 kDa salivary protein	99	Yes
AAEL005056	Electron transfer flavoprotein beta subunit	94	No
AAEL003182	Serine protease inhibitor (serpin)	73	Yes
AAEL012175	F1 ATP synthase alpha subunit	57	No
AAEL006424	D7 protein long	53	Yes
AAEL013144	Translation initiation factor 3 subunit	49	No
AAEL010242	8.7 kDa secreted protein	48	No

^a^ Protein hits represent unique identifiers that were not present in the negative control.

**Table 5:** LC+MS/MS hits from *Aedes aegypti* salivary gland extract.

Identifier	Comment	Score
AAEL009993	SGS1	5240
AAEL009992	N/A	4161
AAEL002827	ATP synthase beta subunit	1343
AAEL000732	62 kDa secreted protein, putative	1238
AAEL003182	serine protease inhibitor (serpin) homologue—unlikely to be inhibitory	1056
AAEL011197	actin	1050
AAEL005961	actin	954
AAEL006347	apyrase precursor	887
AAEL001928	actin-1	880
AAEL009955	vitellogenin-like protein	812
AAEL000749	angiopoietin-like protein splice variant	798
AAEL006333	salivary apyrase, putative	795
AAEL008787	V-type proton ATPase catalytic subunit A	782
AAEL000748	62 kDa secreted protein, putative	778
AAEL001951	actin	771
AAEL005672	adenosine deaminase	737
AAEL002851	tubulin beta chain	731
AAEL012175	ATP synthase alpha subunit mitochondrial	728
AAEL009081	56.5 kDa secreted protein, putative	717
AAEL009185	arginine or creatine kinase	701
AAEL005733	myosin heavy chain, nonmuscle or smooth muscle	629
AAEL013739	electron transport oxidoreductase	614
AAEL006642	tubulin alpha chain	585
AAEL003655	N/A	580
AAEL005656	myosin heavy chain, nonmuscle or smooth muscle	577
AAEL009691	carboxylase:pyruvate/acetyl-coa/propionyl-coa	576
AAEL012576	pyruvate kinase	534
AAEL010697	3-ketoacyl-coa thiolase, mitochondrial	511
AAEL004988	phosphoglycerate kinase	510
AAEL003053	allergen, putative	510
AAEL005766	fructose-bisphosphate aldolase	508
AAEL002704	serine protease inhibitor (serpin) homologue	499
AAEL003872	translationally-controlled tumor protein homolog (TCTP)	467
AAEL010146	3-hydroxyacyl-coa dehyrogenase	440
AAEL011288	elongation factor 1 gamma	432
AAEL007420	serine protease inhibitor (serpin) homologue—unlikely to be inhibitory	429
AAEL003734	aconitase, mitochondrial	428
AAEL006485	inosine-uridine preferring nucleoside hydrolase	417
AAEL004338	pyruvate dehydrogenase	411
AAEL002542	triosephosphate isomerase	411
AAEL001593	glycerol-3-phosphate dehydrogenase	393
AAEL000951	elongation factor 1-beta2	375
AAEL008166	malate dehydrogenase	372
AAEL010821	60S acidic ribosomal protein P0	362
AAEL013614	clathrin heavy chain	353
AAEL005515	heterogeneous nuclear ribonucleoprotein	350
AAEL010235	30 kDa salivary gland allergen Aed a 3 Precursor (Allergen Aed a 3)	349
AAEL006582	calcium-transporting ATPase sarcoplasmic/endoplasmic reticulum type	340
AAEL006719	alpha-amylase I precursor	339
AAEL012827	endoplasmin	330
AAEL011584	chaperonin-60kD, ch60	330
AAEL011704	heat shock protein	328
AAEL003100	salivary mucin	322
AAEL005052	tubulin beta chain	312
AAEL003107	N/A	311
AAEL006417	D7 protein, putative	309
AAEL006833	succinyl-CoA synthetase small subunit, putative	308
AAEL003600	34 kDa salivary protein, putative	306
AAEL004297	ATP-citrate synthase	302
AAEL006721	2-oxoglutarate dehydrogenase	297
AAEL014452	acyl-coa dehydrogenase	296
AAEL004500	eukaryotic translation elongation factor	295
AAEL008167	aspartate ammonia lyase	287
AAEL010585	spermatogenesis associated factor	285
AAEL000793	venom allergen	284
AAEL001194	fatty acid synthase	284
AAEL007555	acyl-coa dehydrogenase	280
AAEL009651	nascent polypeptide associated complex alpha subunit (nac alpha)	270
AAEL002764	dihydrolipoamide succinyltransferase component of 2-oxoglutarate dehydrogenase	269
AAEL013613	pyruvate dehydrogenase	267
AAEL002572	myosin regulatory light chain 2 (mlc-2)	266
AAEL007065	ADP-ribosylation factor, arf	265
AAEL006096	gelsolin precursor	263
AAEL010464	glutamate dehydrogenase	262
AAEL008192	40S ribosomal protein S3	239
AAEL005931	6-phosphogluconate dehydrogenase	237
AAEL013904	3-hydroxyisobutyrate dehydrogenase	234
AAEL002841	3-hydroxyacyl-coa dehyrogenase	233
AAEL005798	ATP synthase subunit beta vacuolar	231
AAEL006834	glutamate semialdehyde dehydrogenase	228
AAEL007707	malate dehydrogenase	225
AAEL013144	eukaryotic translation initiation factor 3 subunit I (eIF3i)	221
AAEL000726	fibrinogen and fibronectin	221
AAEL004294	dihydrolipoamide acetyltransferase component of pyruvate dehydrogenase	208
AAEL003694	N/A	206
AAEL002296	trifunctional enzyme beta subunit (tp-beta)	203
AAEL006040	coatomer	201
AAEL005084	tubulin beta chain	201
AAEL004739	acyl-coa dehydrogenase	199
AAEL007718	eukaryotic translation initiation factor 3 subunit B (eIF3b)	195
AAEL013353	profilin	195
AAEL003530	acidic ribosomal protein P1, putative	190
AAEL000392	maltase-like 1	189
AAEL005422	pyrroline-5-carboxylate dehydrogenase	189
AAEL001588	glutamate carboxypeptidase	184
AAEL008619	trypsin-like salivary secreted protein	183
AAEL003125	acyl-coa dehydrogenase	181
AAEL008844	calcium-binding protein, putative	181
AAEL002693	venom allergen	178
AAEL013359	DEAD box ATP-dependent RNA helicase	176
AAEL002309	thioredoxin peroxidase	174
AAEL009029	aldehyde dehydrogenase	173
AAEL006424	37 kDa salivary gland allergen Aed a 2 precursor (protein D7)(allergen Aed a 2)	170
AAEL003634	hsp70-interacting protein, putative	169
AAEL008006	3-hydroxyacyl-coa dehyrogenase	163
AAEL007945	eukaryotic translation initiation factor 3 subunit H (eIF3h)	161
AAEL009902	eukaryotic translation initiation factor 3 subunit D (eIF3d)	154
AAEL001134	probable methylmalonate-semialdehyde dehydrogenase [acylating], mitochondrial precursor	151
AAEL001668	enolase	147
AAEL011746	succinyl-coa synthetase beta chain	147
AAEL012359	nucleoside-diphosphate kinase NBR-A, putative	143
AAEL003601	34 kDa secreted protein, putative	140
AAEL005069	ras-related protein Rab-1A, putative	139
AAEL009313	elongation factor -1 beta,delta	138
AAEL012746	chaperonin	138
AAEL013675	eukaryotic translation initiation factor	136
AAEL013625	40S ribosomal protein S5	135
AAEL009747	40S ribosomal protein S18	134
AAEL011756	aldehyde dehydrogenase	128
AAEL001005	calreticulin	126
AAEL000641	protein disulfide isomerase	126
AAEL001218	alanyl-tRNA synthetase	126
AAEL001128	AMP dependent coa ligase	126
AAEL010143	isocitrate dehydrogenase	125
AAEL006174	proteasome subunit beta type	123
AAEL012207	myosin light chain 1	122
AAEL008848	ATP synthase gamma subunit	121
AAEL000703	glycogen phosphorylase	121
AAEL006634	acetyl-coa acetyltransferase, mitochondrial (acetoacetyl-coa thiolase)	121
AAEL003993	cyclohex-1-ene-1-carboxyl-CoA hydratase, putative	119
AAEL005567	nucleosome assembly protein	117
AAEL006085	methylenetetrahydrofolate dehydrogenase	117
AAEL008083	40S ribosomal protein SA	114
AAEL004378	eukaryotic translation initiation factor 1A (eIF-1A)	114
AAEL013069	receptor for activated protein kinase c (rack1)	113
AAEL002504	ATP synthase delta chain, mitochondrial	112
AAEL004347	eukaryotic translation initiation factor 3 subunit M (eIF3m)	111
AAEL009617	eukaryotic translation initiation factor 3 subunit L (eIF3l)	107
AAEL012825	bifunctional purine biosynthesis protein	107
AAEL006928	dihydrolipoamide dehydrogenase	106
AAEL002833	cathepsin l	106
AAEL002334	eukaryotic translation initiation factor 3 subunit E (eIF3e)	105
AAEL009872	alanine aminotransferase	105
AAEL011741	glutathione transferase	104
AAEL007064	Gram-negative Binding Protein (GNBP) or beta-1 3-glucan binding protein	104
AAEL005269	ubiquinol-cytochrome c reductase complex core protein	103
AAEL007033	pyrroline-5-carboxylate reductase	103
AAEL005407	annexin x	102
AAEL001331	mannose-1-phosphate guanyltransferase	102

**Fig 6:**
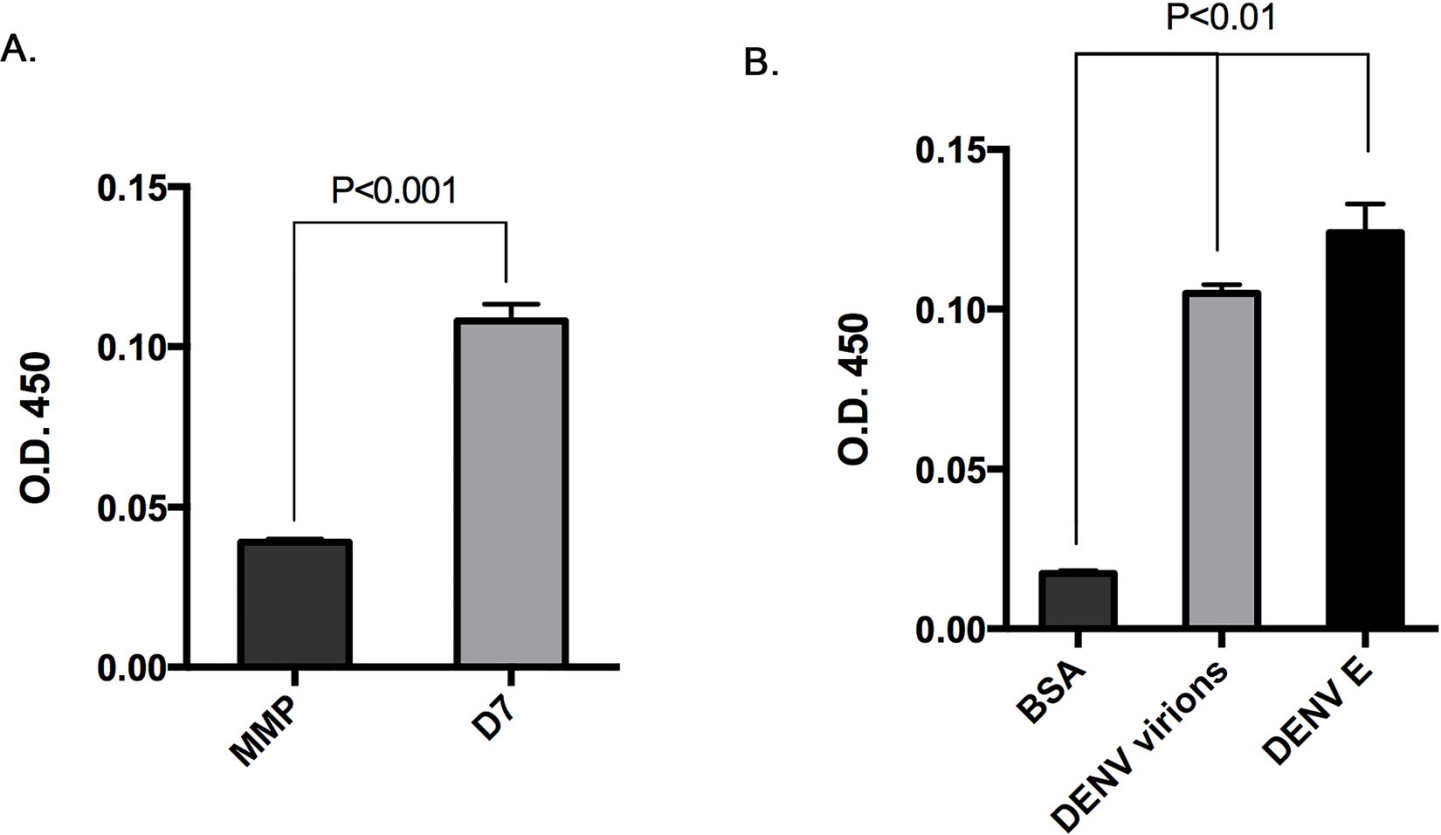
Recombinant D7 protein binds DENV2 virions and DENV2 envelope protein. (A) An enzyme-linked immunosorbent assay was developed by coating plates with control MMP protein or recombinant D7 protein and then measuring binding of DENV2 virions. (B) An enzyme-linked immunosorbent assay was developed by coating plates with recombinant D7 protein and then measuring binding of bovine serum albumin (BSA), DENV2 virions, and recombinant DENV2 envelope protein. Interactions were detected using antibodies against DENV2 envelope protein and a secondary antibody conjugated to horse radish peroxidase. Absorbance was detected at OD 450 nm. Student’s t tests were performed to assess statistical significance between groups.

To confirm if D7 protein can directly interact with DENV2, we performed an enzyme-linked immunosorbent assay (ELISA) by binding a control MMP protein or recombinant D7 protein to a 96 well plate [24]. Plates were then incubated with DENV2 16881 virions. Unbound proteins were then removed by washing each well with buffer. The degree of association between immobilized proteins and DENV2 virions was assessed using an antibody that recognized DENV followed by a secondary antibody conjugated to horse radish peroxidase. DENV2 16881 virions bound to D7 protein more readily than MMP protein. (Fig 6A). Plates coated with D7 protein were then incubated with bovine serum albumin, DENV2 16881 virions, or recombinant DENV2 envelope protein. Unbound proteins were then removed by washing each well with buffer. The degree of association between D7 and the above proteins was assessed using an antibody recognizing DENV followed by a secondary antibody conjugated with horse radish peroxidase. We found that recombinant D7 interacted with both DENV2 16881 virions and recombinant DENV2 envelope protein (Fig 6B).

## Discussion

Hematophagous arthropod saliva contains a complex mixture of proteins with anti-hemostatic, anti-inflammatory, and immunomodulatory properties. Hematophagous arthropod saliva can also enhance transmission of arboviruses, although the exact mechanism and saliva proteins involved in this process are not known [3]. Interestingly, individual saliva components can have inhibitory activities that prevent arbovirus infection [19]. Here, we identified D7 protein in biochemical fractions of salivary gland extracts that inhibited DENV2 cell binding and/or infection in mouse embryonic fibroblasts and then determined if this activity could be recapitulated with recombinant D7 protein. We found that recombinant D7 protein prevented DENV2 cell binding and/or infection in two permissive cell types and prevented infection and dissemination in a mouse model. Additionally, two separate binding assays suggest that D7 protein can physically interact with DENV virions and envelope protein. These data support the previous observation that D7 protein vaccination enhanced mortality in a West Nile virus mouse model and suggest that D7 protein inhibits virus transmission [20].

D7 proteins are some of the most abundant proteins expressed in the salivary glands of blood feeding *Diptera*. Long and short forms of D7 proteins exist in mosquitoes. Each of these proteins appear capable of binding either cysteinyl leukotrienes, and/or biogenic amines such as serotonin, histamine, and norepinephrine. These functions are predicted to antagonize the host’s inflammatory response, and ability to vasoconstrict, induce platelet-aggregation, and induce a sense of pain–each critical to efficiently obtaining a blood meal [21, 22].

It is theoretically possible that the functions of D7 proteins are counter to the needs of arboviruses during transmission to a vertebrate host. D7 proteins bind multiple substrates and it is possible that they bind other proteins with lower affinity, which may limit virus-host interactions. Additionally, limiting the host inflammatory response may reduce influx or activation of target cells, such as Langerhans cells, monocytes, or macrophage. Our data support that D7 protein mediates its antiviral effect through direct protein-protein interaction *in vitro*, although it is possible that modulation of the inflammatory response also occurs *in vivo*.

D7 proteins are some of the most abundant and immunogenic proteins present in mosquito saliva [25]. The presence of anti-D7 antibodies has been used as a marker of exposure to certain mosquito species [26–29]. Considering that individuals who are exposed to mosquitoes have high levels of anti-D7 antibodies, it is likely that these antibodies inhibit D7 protein function. In fact, the presence of anti-D7 antibodies has been linked to disease severity [29]. Although anti-D7 antibodies may prevent efficient blood feeding by a mosquito, it may also enhance disease transmission and disease severity. Characterizing the complex interplay of virus-vector-host interactions will lead to the development of better models of pathogenesis, strategies to limit disease transmission and promote the development of therapeutics, and transmission-blocking vaccines.
